# Supplementary material for: End Sequence Analysis Toolkit (ESAT) expands the extractable information from single-cell RNA-seq data
Source: Genome Res. 2016 Oct;26(10):1397–410. doi: 10.1101/gr.207902.116 (PMC5052061; doi:10.1101/gr.207902.116)
Supplement: Supplemental Material [file supp_gr.207902.116_Supp_Materials.tar.gz › ESAT-esat_v0.1/doc/html/classumms_1_1core_1_1model_1_1score_1_1_length_score.html]

ESAT: umms.core.model.score.LengthScore Class Reference


ESAT


- Main Page
- Related Pages
- Packages
- Classes
- Files

- Class List
- Class Index
- Class Hierarchy
- Class Members

All Classes Namespaces Files Functions Variables Enumerator Pages

- umms
- core
- model
- score
- LengthScore

Classes |
Public Member Functions |
List of all members

umms.core.model.score.LengthScore Class Reference

Inheritance diagram for umms.core.model.score.LengthScore:

[legend]

Collaboration diagram for umms.core.model.score.LengthScore:

[legend]

|  |  |
| --- | --- |
| Classes | |
| class | **Processor** |
|  | |

|  |  |
| --- | --- |
| Public Member Functions | |
|  | LengthScore (Annotation a) |
|  | |
|  | LengthScore (AnnotationCollection<?extends Annotation > model, Annotation a) |
|  | |
| Public Member Functions inherited from umms.core.model.score.AbstractListScore | |
|  | AbstractListScore (Annotation a) |
|  | |
| void | setScores (List< Double > scores) |
|  | |
| double | getScore () |
|  | |
| double | getCount () |
|  | |
| double | getSum () |
|  | |
| double | getMedian () |
|  | |
| double | getMean () |
|  | |
| double | getStandardDeviation () |
|  | |
| double[] | getScores () |
|  | |
| String | toString () |
|  | |
| Public Member Functions inherited from umms.core.model.score.WindowScore.AbstractWindowScore | |
|  | AbstractWindowScore (Annotation t) |
|  | |
| Annotation | getAnnotation () |
|  | |

|  |  |
| --- | --- |
| Additional Inherited Members | |
| Static Public Attributes inherited from umms.core.model.score.AbstractListScore | |
| static double | DEFAULT\_VALUE = -1.0 |
|  | |
| Protected Attributes inherited from umms.core.model.score.WindowScore.AbstractWindowScore | |
| Annotation | annotation |
|  | |

## Constructor & Destructor Documentation

|  |  |  |  |  |  |
| --- | --- | --- | --- | --- | --- |
| umms.core.model.score.LengthScore.LengthScore | ( | Annotation | *a* | ) |  |

|  |  |  |  |
| --- | --- | --- | --- |
| umms.core.model.score.LengthScore.LengthScore | ( | AnnotationCollection<?extends Annotation > | *model*, |
|  |  | Annotation | *a* |
|  | ) |  |  |

Here is the call graph for this function:

---

The documentation for this class was generated from the following file:

- java/umms/core/model/score/LengthScore.java


---

Generated on Fri Aug 15 2014 15:11:12 for ESAT by  

 1.8.7
